# Role of Galectins in Multiple Myeloma

**DOI:** 10.3390/ijms18122740

**Published:** 2017-12-17

**Authors:** Paola Storti, Valentina Marchica, Nicola Giuliani

**Affiliations:** 1 Department of Medicine and Surgery, University of Parma, Via Gramsci, 14, 43126 Parma, Italy; paola.storti@unipr.it (P.S.); valentina.marchica@studenti.unipr.it (V.M.); 2 Hematology, “Azienda Ospedaliero-Universitaria di Parma”, Via Gramsci, 14, 43126 Parma, Italy

**Keywords:** galectins, myeloma, galectin-1, galectin-3, galectin-8, galectin-9

## Abstract

Galectins are a family of lectins that bind β-galactose-containing glycoconjugates and are characterized by carbohydrate-recognition domains (CRDs). Galectins exploit several biological functions, including angiogenesis, regulation of immune cell activities and cell adhesion, in both physiological and pathological processes, as tumor progression. Multiple myeloma (MM) is a plasma cell (PC) malignancy characterized by the tight adhesion between tumoral PCs and bone marrow (BM) microenvironment, leading to the increase of PC survival and drug resistance, MM-induced neo-angiogenesis, immunosuppression and osteolytic bone lesions. In this review, we explore the expression profiles and the roles of galectin-1, galectin-3, galectin-8 and galectin-9 in the pathophysiology of MM. We focus on the role of these lectins in the interplay between MM and BM microenvironment cells showing their involvement in MM progression mainly through the regulation of PC survival and MM-induced angiogenesis and osteoclastogenesis. The translational impact of these pre-clinical pieces of evidence is supported by recent data that indicate galectins could be new attractive targets to block MM cell growth in vivo and by the evidence that the expression levels of *LGALS1* and *LGALS8*, genes encoding for galectin-1 and galectin-8 respectively, correlate to MM patients’ survival.

## 1. Biological and Pathophysiological Functions of Galectins

### 1.1. Galectin Family

Galectins are a family of lectins, evolutionarily conserved and with the ability to bind glycans [1]. All galectins contain one or more carbohydrate-recognition domains (CRDs), sequences of about 130 amino acids, responsible for the binding to carbohydrate [1,2]. To date, 15 mammalian galectins have been identified and some seem to be species specific, such as galectin-5 and galectin-6 found only in rodents, and galectin-11 and galectin-15 only in caprine and ovine [2,3]. Based on the number and the structure of CRD, galectins are divided in three groups: prototype (galectin-1, -2, -5, -7, -10, -11, -13, -14 and -15) that have only a CRD that can associate in dimer, tandem repeat-type (galectin-4, -6, -8 and -9) that carry two CRDs linked by a short peptide linker, and chimera-type (galectin-3) that has a CRD connected to a non-lectin amino-terminal region that allow the oligomerization into pentamers [1,2,4].

Galectins can be found in the intracellular compartment and in the cell nuclei; moreover, several galectins are secreted by cells through a non-classical secretory mechanism, lacking the signal sequence for classical secretion, and they are detected in the extracellular space [1,5]. Extracellularly, galectins can bind to cell surface glycoconjugates, bearing the *N*-acetyl-lactosamine (Galβ(1-4)GlcNAc; LacNAc) disaccharide, forming a galectin–glycan structure called lattice, and mediating an intracellular signal transduction [1,6,7,8]. Moreover, they can bind to some glycoproteins of the extracellular matrix, such as laminin, fibronectin and elastin [9]. In fact, each member of the galectin family exhibits preferences in different glycan binding [10], which could explain their differences in biological and pathophysiological functions and the wide range of identified receptors on cell surfaces [2,11]. In particular, the tandem repeat type galectins, galectin-8 and -9, carry two CRDs (N and C terminal) that, despite their structural similarity, recognize different oligosaccharides (i.e., sulfated or sialylated glycans or biantennary oligosaccharide) due to different affinity [12,13,14]. Moreover, the modification of the short peptide linker of tandem repeat-type galectins could also modify their biological functions [15]. Indeed, galectins could crosslink different glycoconjugates and trigger a cascade of transmembrane signaling pathways or they can cause the clustering of multiple glycoconjugates on cell surfaces [7].

The expression and the function of the galectins involved in bone marrow (BM) microenvironment are summarized in Table 1.

### 1.2. Galectins in Hematopoiesis and Immunity

Galectins have a role in hematopoietic differentiation, in particular creating specific galectin–glycan interactions between hematopoietic and stromal cells, sustaining the formation of a microenvironmental niche [46].

During the T cell development, in the thymus, galectin-1, -3, -8 and -9 induce the apoptosis of the double positive (CD4+CD8+) or double negative (CD4−CD8−) developing thymocytes, favoring the interaction between thymocytes and thymic epithelial cells [46]. Once in the periphery, galectins fine regulate T cells homeostasis. Galectin-1 prolongs the survival of T naive cells, induces the apoptosis of T helper (Th) 1 and Th17 differentiated cells and protects Th2 cells, promoting the release of anti-inflammatory cytokines (interleukin (IL)-4, IL-5 and IL-10) [11]. Galectin-9 interacts with its receptor T-cell immunoglobulin and mucin-domain containing-3 (TIM-3) on Th1 cell surface, leading to their apoptosis [47]. Galectin-1 and -9 induce the expansion of FoxP3+ T regulatory (Treg) cells.

In B cell differentiation, galectin-1 creates a synapse between pre-B cell receptor (pre-BCR) on preB-cells and BM stromal cells, leading pre-BCR clustering and signaling [48]. In the periphery, galectin-1 is upregulated in PCs by the activation of B-lymphocyte-induced maturation protein (BLIMP)-1 and, with galectin-8, promotes the immunoglobulin production [11,18]. On the other hand, intracellular galectin-3 favors the differentiation toward memory B cells [46].

Thereafter, galectins regulate antigen presenting cell functions; in particular, galectin-1 induces the dendritic cell (DC) tolerogenic phenotype and the macrophage switch to M2 phenotype [49] and galectin-9 stimulates DCs and activates innate immunity [50].

Finally, galectin-3 is also involved in bone homeostasis. Galectin-3 deficient mice display increased osteoclast (OC) activity and, in vitro, galectin-3 interferes with the receptor activator of nuclear factor kappa-B ligand (RANKL) signaling on OCs, reducing their differentiation [32].

### 1.3. Galectins and Tumor Progression

Galectins are involved, since the first phases of tumor transformation, in the survival of tumoral cells [51]. Based on literature data, the inhibition of galectin-1 and -3 expression reverts the transformed phenotype into normal in glioma [52], breast [53] and thyroid papillary carcinoma cells [33]. Moreover, galectin-1 and -3 can interact with the oncogenic Ras proteins that are anchored to the cellular membrane [54], and sustain the activation of their downstream effectors, such as extracellular signal-regulated kinases (ERK), Raf-1 proto-oncogene, serine/threonine kinase (RAF1) and phosphoinositide 3-kinase (PI3K) [51,54,55].

Furthermore, galectins have a role in the regulation of cancer cells apoptosis and cell cycle. Different studies highlighted that galectin-1 could have different effects on cancer cell proliferation, depending on the localization of this lectin (intracellular or extracellular) and on the tumor type [29].

Moreover, galectin-1, -3 and -8 can support tumor cells migration and attachment to extracellular matrix (ECM) [56,57] but the major role of galectin in the tumor progression is their interaction with the microenvironment, promoting neo-angiogenesis and inducing immune escape. Galectin-1 promotes endothelial cell (EC) proliferation and migration, thus angiogenesis, binding to the vascular endothelial growth factor receptor (VEGFR) 2 and neuropilin-1. In Kaposi’s sarcoma, prostate cancer, lung cancer and melanoma [17,58,59], it mimics the effect of vascular endothelial growth factor A (VEGFA) and confers resistance to anti-VEGF therapy [58]. Galectin-3 and -8 support the vascularization process in the tumor microenvironment binding to integrin α_v_β_3_ and activated leucocyte cell adhesion molecule (ALCAM), respectively [39,60].

### 1.4. Galectins and Tumoral Immune Microenvironment

Tumor immune microenvironment is heavily shaped by galectins. One of the effects of galectins is to expand the regulatory myeloid cells; in fact, galectin-1 promotes differentiation of IL-27- and IL-10-producing tolerogenic DCs [61], and contributes to M2 macrophage polarization [49]. Galectin-1 and -9 also support the recruitment of myeloid-derived suppressor cells (MDSCs) and increase their regulatory capacity [16,43]. Secondly, galectin-1, -8, -9- and -10 enhance the expansion of Tregs and increase the immunosuppressive activity of CD4+CD25+Foxp3+ Tregs [62,63]. Further, galectin-1 and -9 selectively induce apoptosis in Th1 and Th17 effector cells [11]. On the other hand, galectin-3 and -9 participate in the immune inhibitory checkpoints cascade, including interactions with lymphocyte activation gene (LAG)-3 or TIM-3 [34,47], leading to an inhibition of tumor infiltrating T cells. Finally, in glioblastoma, galectin-1 induces a natural killer (NK) cell inhibition, mediated by the increased activity of tumor related immunosuppressive MDSCs [64]. In addition, galectin-3 and -9 reduce NK cell activity and cytokine production [63]. All the mechanisms described above decrease the potential of anti-tumor immune cells and boost the tumor immune evasion [63].

### 1.5. Galectins and Hematological Malignancies

In hematological malignancies, increased galectin-1 serum levels are correlated with increased tumor burden in Hodgkin lymphoma patients [65] and this lectin is highly expressed in cutaneous T-cell lymphomas cells [21] and chronic lymphocytic leukemia patients [66]. In Hodgkin lymphomas, galectin-1 reduces anti-tumor T cell activity, promoting the expansion of CD4+CD25+Foxp3+ Tregs [67]. Moreover, in lymphoma, galectin-1 expression is strongly correlated with resistance to immunotherapy; in fact, galectin-1-overexpressing lymphoma cells blunt antibody-dependent tumor phagocytosis in vitro, and, in vivo, galectin-1 reduces lymphoma cells sensitivity to CD20 immunotherapy [20].

Galectin-3 is overexpressed in diffuse large B-cell lymphoma [68] and chronic myelogenous leukemia [31]. This lectin increases the proliferative and chemotactic capacity of lymphoma and leukemia cells and enhances their resistance to chemotherapy. Finally, in acute myelogenous leukemia, galectin-3 expression is an independent unfavorable prognostic factor for patients’ overall survival (OS) [69] and, in the same disease, galectin-9 induces, through TIM-3, T cell dysregulation [40].

## 2. Multiple Myeloma (MM) Pathophysiology

Multiple myeloma (MM) is a hematological malignancy characterized by an accumulation of malignant plasma cells (PCs) with a tight dependence to the BM microenvironment [70]. The active stage of MM is preceded by indolent stages as monoclonal gammopathy of undetermined significance (MGUS) and smoldering myeloma (SMM) [71]. The progression from an indolent stage to the symptomatic one is supported by sequential genetic events in the malignant clones and by microenvironment alterations that support the growth of malignant PCs [72], as the angiogenic switch and the development of osteolytic lesions [73].

### 2.1. Deregulated Pathways in Malignant Plasma Cells (PCs)

The MM PCs accumulate several genetic lesions, such as translocations, mutations, deletions or amplifications, which lead to a deregulation of different proliferative pathways [72]. In MM, the major translocations involve the immunoglobulins loci, putting under control of a strong enhancer different oncogenes such as cyclin (CCN) D1, CCND3, FGFR3, multiple myeloma SET domain (MMSET), MYC, MAF and MAFB [74]. A later genetic event is the mutation and the monoallelic deletion of chromosome the locus 17p13, carrying the onco-suppressor gene p53 [71]. Other recurrent mutations involve genes of ERK pathway (NRAS, BRAF and KRAS) [72] and of nuclear factor- kappa B (NF-κB) pathway (CYLD, TRAF3 and BIRC2/3) [75].

The malignant PCs are dependent on the BM microenvironment and the interaction between MM PCs and BM cells occurs mainly through adhesion molecules, chemokines and growth factors that support the survival and the proliferation of the malignant clones [73]. The PCs adhere to ECM by molecules, such as fibronectin and type I collagen, and the bone marrow stromal cells (BMSCs) interact with the malignant PCs using different molecular complexes as very late antigen-4 (VLA-4)/vascular cell adhesion molecule (VCAM)-1, CD38/CD31, lymphocyte function associate antigen (LFA)-1/intercellular adhesion molecule (ICAM)-1 and the homotypic binding of CD56 [76,77]; all the interactions described above support the production of soluble factors that sustain the growth of MM PCs. The major growth factor of MM cells is IL-6, produced either from PCs or BMSCs or osteoblasts (OBs); subsequently, the stimulation of NF-κB pathway activates the mitogen-activated protein kinase kinase 1 (MEK)/ERK, Janus kinase/signal transducers and activators of transcription (JAK/STAT3) and PI3K/ Protein kinase B (AKT) signaling pathways that promote cell survival and apoptosis resistance [78,79]. Besides IL-6, other important pro-survival and proliferation cytokines are VEGFA, insuline-like growth factor (IGF)-1, tumor necrosis factor α (TNFα), transforming growth factor β (TGFβ) and IL-1β [77,80].

### 2.2. Microenvironment Alterations in MM: Role of Angiogenesis and Bone Destruction

The MM progression is characterized by an avascular phase, corresponding to the two indolent stages SMM and MGUS, followed by an angiogenic switch, leading to the active MM [81]. Moreover, the BM microenvironment is hypoxic, and the MM cells overexpress the main factor involved in the cell adjustment to hypoxic stress, the hypoxia inducible factor (HIF)-1α [82,83]. Indeed, the MM angiogenic switch is supported by BM hypoxia, HIF-1α and the production of pro-angiogenic molecules either by MM PCs or by BMSCs, such as VEGFA, fibroblast growth factor (FGF), hepatocyte growth factor (HGF), angiopoietin (ANG)-1 and osteopontin (OPN), which act as chemoattractants or bind to receptor on endothelial cells (ECs), such as tyrosine kinase with immunoglobulin-like and EGF-like domains (TIE)-2 and VEGFR2, and promote their proliferation [81,84,85]. Moreover, BMSCs and MM cells secrete the proteolytic enzymes metalloprotease (MMP)-1, MMP-2 and MMP-9 that help to reshape the extracellular compartment and the migration of ECs [86]. The interaction between MM cells and ECs, besides supports the angiogenic process, favors the homing of MM PCs into the BM supporting their survival [87].

One of the features of active MM is the presence of bone lesions, due to an imbalance between OC and OB formation and activity [88]. In the MM BM microenvironment, the RANKL/osteoprotegerin (OPG) ratio is altered; in particular, MM cells upregulate the production of RANKL and downregulate OPG expression by BMSCs and T lymphocytes [89,90]. Moreover, MM PCs sustain the osteoclastogenesis and the OC activity overexpressing chemokine (C-C motif) ligand (CCL)-3, IL-3, and IL-7 [91] [92]. Furthermore, activin A, secreted by BMSCs and monocytes, promotes OC differentiation and OB inhibition [93]. CCL20 is an additional factor involved in MM-induced OC activity [94]. Thereafter, MM cells interact with BMSCs and pre-OBs, through VLA-4/VCAM-1 and CD56/CD56 binding, and, in the latter ones, suppress the activity of the main pro-osteoblastogenic transcription factor, Runt-related transcription factor (Runx)-2, leading to an inhibition of OB differentiation [95]. Runx2 activity is also reduced by other soluble cytokines secreted by MM cells, as IL-7, IL-3 and HGF [96]. Finally, MM cells also inhibit the receptor for Wnt family member (Wnt)-5a, receptor tyrosine kinase-like orphan receptor (ROR)-2, on OB precursors and, indeed, block the activation of non-canonical Wnt signaling, important for OB differentiation [97].

### 2.3. The Immune Microenvironment in MM

MM patients share also several alterations in the immune system, due to a deficit of humoral immunity, immunoparesis and alterations in the activity of effectors cells [98]. In MM patients’ peripheral blood, it is reported a reduction of the CD4+/CD8+ T cells ratio and an imbalance between Th1 and Th2, due to an overproduction of pro-Th2 cytokines, such as IL-4 and IL-10 [99]. In addition, an increase of Th17 cells is reported in MM BM; these cells secrete IL-17 that suppresses cytotoxic T cell activity, supports PC growth and is a key mediator of MM bone disease [100,101]. Moreover, MM cells express programmed cell death-ligand (PD-L)-1 that binds its receptor programmed cell death (PD)-1 expressed by T and NK cells and is deeply involved in the immunosuppression that characterize the MM immune microenvironment [102]. DCs are also dysfunctional for a deficit of co-stimulatory molecules, such as CD80 and CD86, needed for the activation of T cells [103]. The lack of DC functional activity is due to an accumulation of cytokines in the MM BM microenvironment, such as IL-6, IL-10, TGFβ and VEGFA [104]. Moreover, the interaction between MM PCs and DCs increases the production of indoleamine-pyrrole 2,3-dioxygenase (IDO) that promotes the T anergic phenotype and Tregs differentiation [105]. Finally, MM patients have elevated levels of MDSCs, compared to healthy subjects [106], and they are involved in the immune escape of MM cells.

## 3. Galectins and Multiple Myeloma

### 3.1. Galectin-1 and MM

In MM cells, galectin-1 is expressed at high level, at both mRNA and protein levels, maintaining inter-patient and inter-human myeloma cell line (HMCLs) variability [23,24]. Moreover, analyses of gene expression datasets of MM primary CD138+ cells revealed that *LGALS1* (galectin-1 gene) levels are significantly higher in newly diagnosed MM (MMD) patients, but not in MGUS, SMM and MM relapsed, compared to healthy donors [24,107]. Recently, it has been published that, in peripheral blood sera, galectin-1 protein level was borderline significantly higher in MMD compared to healthy controls and that the levels of this lectin in peripheral blood are not associated with OS, response to treatment and clinical pathological parameters [108]; on the other hand, this study shows only a positive correlation between galectin-1 and soluble (s)CD163, a macrophage activation marker, and sCD138 [108]. Moreover, galectin-1 has been identified as a ECM-associated protein that characterizes only the MM BM and not the ECM of MGUS patients or healthy controls [107].

**Figure 1:**
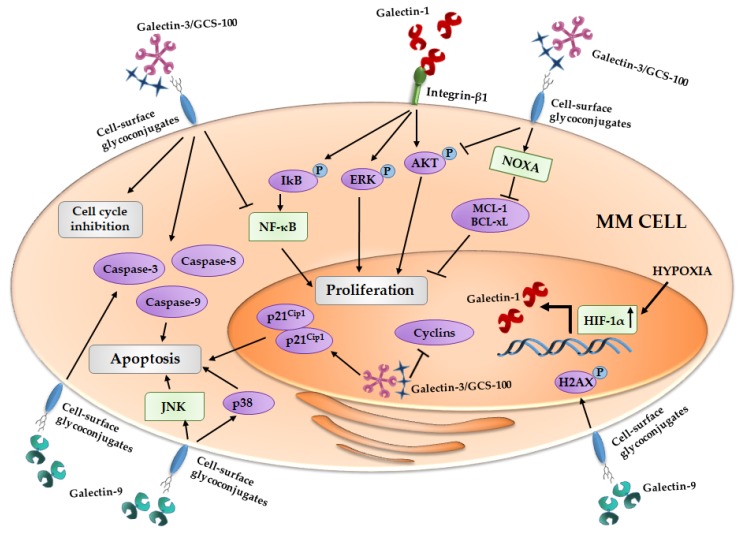
Effect of galectins on MM cells proliferation and apoptosis induction. Galectin-1 is regulated by HIF-1α and the galectin-1/integrin β1 binding induces pro-survival cascades. The inhibition of galectin-3 by GCS-100 (reported in figure as Galectin-3/GCS-100) leads to cell cycle arrest and an activation of different pro-apoptotic signals. Galectin-9 exerts its anti-proliferative activity inducing pro-apoptotic pathways. MM, multiple myeloma; IkB, inhibitor of kappa B; ERK, Extracellular signal-regulated kinases; AKT, Protein kinase B; NOXA, Phorbol-12-myristate-13-acetate-induced protein 1; MCL-1, Induced myeloid leukemia cell differentiation protein Mcl-1; Bcl-xL, B-cell lymphoma-extralarge; NF-κB, nuclear factor-kappaB; HIF-1α, Hypoxia inducible factor-1α; H2AX, H2A histone family, member X; JNK, c-Jun N-terminal kinases.

Thereafter, Panero et al. highlighted that overexpression of *LGALS1* is associated with high mRNA expression of telomerase (*hTERT*) MM cells, ascribing a role of these lectin in MM cell proliferation [109]. In line with these data, in the first evidence of the role of galectin-1 in MM cells, Abroun et al. reported that galectin-1 binds β1-integrin and supports the proliferation of CD45RA(−) HMCLs, increasing the phosphorylation of ERK, AKT and IkB, and it has an opposite effect on CD45RA(+) MM cells, reducing their proliferation [23] (Figure 1).

**Figure 2:**
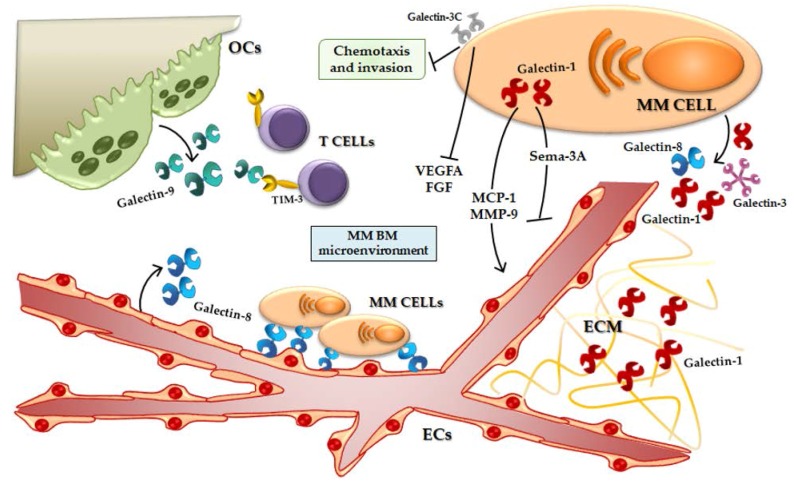
Galectins in the MM BM microenvironment. Galectin-1 induces the secretion of pro-angiogenic molecules and inhibits the production of anti-angiogenic ones. Galectin-1 is present in the ECM of MM patients. Galectin-3C reduces MM cell chemotaxis and invasion and the secretion of pro-angiogenic proteins. Galectin-8 is produced by ECs and mediates the adhesion between MM PCs and ECs. Galectin-9 is secreted by OCs and could mediate the T cell activity inhibition. OCs, Osteoclasts; TIM-3, T-cell immunoglobulin and mucin-domain containing-3; MM, multiple myeloma; VEGFA, Vascular endothelial growth factor *A;* FGF, Fibroblast growth factor; MCP-1, Monocyte chemoattractant protein-1; MMP-9, Metalloprotease-9; Sema-3A, Semaphorin -3A; ECM, Extracellular matrix; ECs, Endothelial cells; BM, bone marrow.

Recently, our group demonstrated that, in MM cells, galectin-1 is upregulated by hypoxia and its expression is controlled by HIF-1α [24]. Moreover, in HMCLs, the suppression of *LGALS1* by a shRNA lentiviral vector does not induce an alteration of cell proliferation or survival, but, on the other hand, galectin-1 inhibition leads to downregulation of pro-angiogenic molecules, such as monocyte chemoattractant protein (MCP)-1 and MMP-9, and an up-regulation of anti-angiogenic ones, such as Semaphorin-3A [24]. Indeed, galectin-1 suppression reduces the pro-angiogenic properties of HMCLs conditioned media (CM) in in vitro vessels formation [24] (Figure 2).

Finally, two different in vivo mouse models demonstrated the role of galectin-1 as a putative target in MM [24]. In both models, galectin-1 inhibition in MM cells significantly reduces tumor masses, tumor angiogenesis, and, in the intratibial model, the formation of bone lesions [24].

### 3.2. Galectin-3 and MM

Galectin-3 is variable expressed in HMCLs and in about 25% of primary MM CD138+ cells [30,36,110]. A citrus-derived polysaccharide inhibitor of galectin-3, GCS-100, induces apoptosis in primary MM cells and HMCLs, reduces MM cell proliferation supported by adhesion to BMSCs and blocks HMCLs migration induced by VEGFA [36]. In particular, GCS-100 modifies the MM cell cycle, leading to an accumulation of cells in sub-G_1_ and G_1_ phases, and induces an upregulation of the cell cycler inhibitor p21^Cip1^ and a reduction of different cyclins [35]. Thereafter, GCS-100-induced MM cell apoptosis is mediated by a reduction of MCL-1 and Bcl-xL (B-cell lymphoma-extralarge) proteins, an induction of NOXA (Phorbol-12-myristate-13-acetate-induced protein 1) and the activation of caspase-3, -8 and -9, associated with lower levels of activated AKT and NF-κB pathways [35] (Figure 1). Moreover, in MM cells, GCS-100 overcomes resistance to the proteasome inhibitor, bortezomib, and increases the apoptosis induced by dexamethasone treatment [36].

Mirandola et al. studied another galectin-3 negative dominant inhibitor, galectin-3C, a N-terminally truncated form of galectin-3, as a MM putative treatment [110]. Galectin-3C has a mild direct anti-proliferative effect on MM cells but shows an inhibition of chemotaxis and invasion stromal derived factor (SDF)-1α-mediated of U266, a HMCL [110]. Moreover, galectin-3C acts synergistically with bortezomib, reducing the migration of ECs mediated by MM cell CM and lowering secretion of VEGFA and FGF by HMCLs [110] (Figure 2). Finally, in a non-obese diabetic/severe combined immunodeficiency (NOD/SCID) murine model, MM tumor growth was inhibited by galectin-3C administration and, also, galectin-3C treatment enhances the anti-tumoral effect of bortezomib in vivo [110].

### 3.3. Galectin-8 and MM

Recently, Friedel et al. demonstrated that ECs produce galectin-8, in particular the splicing variants Gal-8S and Gal-8L, and HMCLs only the Gal-8S isoform; moreover, they reported that galectin-8 is present in the sera of about 45% of MM patients [38]. Both galectin-8 isoforms bind to MM cells and Gal-8L induces MM cell adhesion to ECs stronger than Gal-8S both in static tests and under dynamic shear stress test [38]. Finally, galectin-8 exploits its role in MM cell adhesion on ECs even after pro-inflammatory stimulus (TNF-α) [38] (Figure 2).

### 3.4. Galectin-9 and MM

Galectin-9, in contrast, has an anti-proliferative effect on cancer cells. In fact, recombinant protease resistant galectin-9 (hGal-9) exerts an anti-proliferative effect on HMCLs, with its efficiency positively correlated with the affinity for hGal-9 of each HMCLs [41]. In MM cells, the apoptotic induction by hGal-9 is mediated by the activation of caspase-3, -8 and -9 and the loss of mitochondrial outer membrane potential [41]. Moreover, the HMCL treatment with hGal-9 causes an up-regulation of c-Jun and JunD, the phosforilation of H2AX protein and the activation of JNK and p38/MAPK pathways that, taken together, support the pro-apoptotic process [41] (Figure 1). hGal-9 exploits its pro-apoptotic effect also on primary CD138+ from MM patients with poor cytogenetics factors and on bortezomib resistant HMCLs [41]. Finally, in in vivo mouse model, MM tumor growth is also inhibited with hGal-9 treatment [41].

Besides, An et al. explored the role of galectin-9 in osteoclastogenesis and MM BM immune suppression [42]. They have shown that galectin-9 protein is expressed by mature OCs but not by OC-precursors (monocytes) or by MM cells, that it is overexpressed during osteoclastogenesis and further induced by interferon-γ (IFN-γ) OC treatment [42]. Thereafter, BM plasma from MM patients show significantly higher levels of galectin-9, compared to healthy controls [42]. The authors concluded indicating a putative role of OC-secreted galectin-9 in Th1 cells negative regulation, through the interaction with its receptor TIM3 on T cell surface [42] (Figure 2).

### 3.5. Translational Implications in MM

As previously discussed, galectins are known to be involved in multiple biological processes and they could be an attractive therapeutic target in MM, blocking tumoral growth. However, there are some evidences that MM cells may change their glycome during the malignant transformation from normal PCs; this aspect deserve to be considered in a possible therapeutic context because changes on some tumor glycoproteins on MM cells surfaces, such as mucin-1, could lead to different affinity to galectins, resulting in possible alterations in their signals activation [111,112].

Targeting galectin-1 by specific inhibitors, e.g., OTX-008 tested in solid tumors [113], could be a strategy to block the process of neo-angiogenesis induced by MM cells, the expansion of the malignant clone and indeed the formation of lytic lesions [23,24]. Another way to reduce the cross talk between MM cell and ECs in the BM, and consequently the angiogenic process, is to disrupt the functions of galectin-8 [38]; however, this mechanism needs further studies since there are few literature data in MM setting. Moreover, the two compounds GCS-100 and Galectin-3C, which target galectin-3, showed promising data in vitro and in vivo, inhibiting MM cells proliferation and overcoming drug resistance [35,36,110]. In addition, galectin-9, that shows a pro-apoptotic effect on MM cells [41], could be targeted through the development of stable galectin-9, to be delivered on MM cells. Finally, because galectin-1, -3 and -9 have a role in tumor immune escape [11], we can suppose that targeting these galectins may restore the immune control of the disease. These aspects should be expanded and the combination of galectin-inhibition with the blocking of immune inhibitory checkpoints cascade by monoclonal antibodies (e.g., Pembrolizumab, Nivolumab or Ipilimumab) or with immunomodulatory drugs (IMiDs) such as lenalidomide and pomalidomide, might deserve further studies.

### 3.6. MM Patients’ Overall Survival and Galectins

Galectins could be also associated with patients’ outcome. In fact, *LGALS1* gene is included in the 70 genes PCs signature of Shaughnessy et al. that defines MM high risk [114]. Moreover, analyses of two independent gene expression datasets reveled that high level of *LGALS1* expression in MM CD138+ cells is correlated with reduced OS in MM patients compared to those with lower level of *LGALS1* [24,107]. In addition, *LGALS1* expression is also involved in patients’ drug resistance; in fact, this gene is included in a 23 genes signature that distinguishes bortezomib-resistant MM mouse cell line from bortezomib-sensible cell line and significantly predicts differences in patient’s outcomes after treatment with bortezomib [115].

Finally, galectin-8 could also be correlated with patients’ outcome: OS and event free survival of MM patients of the total therapy 2 (TT2) and TT3 studies are longer in patients expressing low levels of *LGALS8* (galectin-8 gene) as compared to the *LGALS8* high group [38].

## 4. Conclusions

As we have explored in this review, galectins have pleiotropic effects in MM BM microenvironment, playing a role in survival, apoptosis, angiogenic properties of MM cell and in OC-mediated immunosuppression. Based on these literature data, the galectin-driven processes, which support MM cells survival and disease progression, deserve further studies and specific galectin inhibitors or activators (in the case of galectin-9) should be designed and fine-tuned. Moreover, different studies have demonstrated the relationship between the gene expression profile of *LGALS1* and *LGALS8* with the survival of MM patients indicating a possible prognostic role of these genes. Clearly further prospective studies should be necessary to confirm these observations.

## Figures and Tables

**Table 1:** Sources and functions of the mains galectins.

Galectin	Gene	Sources	Functions	References
Galectin-1	*LGALS1*	Pre-B cells, PCs, ECs, BMSCs, OBs, T cells, NK cells, DCs	Survival, Angiogenesis, Adhesion and migration, Immunosuppression, Invasion metastasis, Drug resistance	[16,17,18,19,20,21,22,23,24,25,26,27,28,29]
Galectin-3	*LGALS3*	OBs, OCs, BMSCs, PCs, T cells, ECs	Adhesion and migration, Angiogenesis, Anti-apoptotic, Invasion metastasis, Regulation bone homeostasis, Drug resistance	[30,31,32,33,34,35,36,37]
Galectin-8	*LGALS8*	ECs, PCs	Angiogenesis Adhesion and migration	[18,38,39]
Galectin-9	*LGALS9*	DCs, ECs, T cells, OCs	Pro-apoptotic, Adhesion, OC differentiation, Immunosuppression	[40,41,42,43]
Galectin-10	*CLC*	eosinophils and basophils	Immunosuppression	[44,45]

PCs, plasma cells; ECs, endothelial cells; BMSCs, bone marrow stromal cells; OBs, osteoblasts; NK, natural killer; DCs, dendritic cells; OCs, osteoclasts

## Abbreviations

ALCAM	Activated Leucocyte Cell Adhesion Molecule
AKT	Protein kinase B
ANG-1	Angiopoietin-1
Bcl-XL	B-cell lymphoma-extralarge
BLIMP-1	B-Lymphocyte-Induced Maturation Protein 1
BM	Bone Marrow
BMSC	Bone Marrow Stromal Cell
CCL	Chemokine (C–C motif) Ligand
CCN	Cyclin
CM	Conditioned Media
CRD	Carbohydrate-Recognition Domains
DC	Dendritic Cell
EC	Endothelial Cell
ECM	Extracellular Matrix
ERK	Extracellular Signal-Regulated Kinases
FGF	Fibroblast Growth Factor
hGal-9	Recombinant Protease Resistant Galectin-9
H2AX	H2A histone family, member X
HIF-1α	Hypoxia inducible factor-1α
HGF	Hepatocyte Growth Factor
HMCL	Human Myeloma Cell Line
hTERT	Telomerase
ICAM-1	Intercellular Adhesion Molecule 1
IDO	Indoleamine-Pyrrole 2,3-Dioxygenase
IFN-γ	Interferon-γ
IGF-1	Insuline-Like Growth Factor 1
IκB	inhibitor of kappa B
IL	Interleukin
JAK	Janus Kinase
JNK	c-Jun N-terminal kinases
LAG-3	Lymphocyte-Activation Gene 3
LFA-1	Lymphocyte Function Associate Antigen 1
MCL-1	Induced myeloid leukemia cell differentiation protein Mcl-1
MCP-1	Monocyte Chemoattractant Protein-1
MDSC	Myeloid-Derived Suppressor Cells
MEK	Mitogen-Activated Protein Kinase Kinase 1
MGUS	Monoclonal Gammopathy of Undetermined Significance
MM	Multiple Myeloma
MMD	Newly Diagnosed Myeloma
MMP	Metalloprotease
MMSET	Multiple Myeloma SET Domain
NF-κB	Nuclear Factor-kappa-B
NK	Natural Killer
NOXA	Phorbol-12-myristate-13-acetate-induced protein 1
OB	Osteoblast
OC	Osteoclast
OPG	Osteoprotegerin
OPN	Osteopontin
OS	Overall Survival
PC	Plasma Cells
PD-1	Programmed Cell Death-1
PD-L1	Programmed Cell Death Ligand -1
PI3K	Phosphoinositide 3-Kinase
pre-BCR	Pre-B Cell Receptor
RAF1	Raf-1 Proto-Oncogene, Serine/Threonine Kinase
RANKL	Receptor Activator of Nuclear Factor kappa-B Ligand
ROR2	Receptor Tyrosine Kinase-Like Orphan Receptor 2
RUNX2	Runt-Related Transcription Factor 2
Sema-3A	Semaphorin-3A
SMM	Smoldering Myeloma
STAT	Signal Transducers and Activators of Transcription
TGFβ	Transforming Growth Factor β
Th	T Helper
TIE-2	Immunoglobulin-like and EGF-like Domains
TIM-3	T-cell Immunoglobulin and Mucin-Domain Containing-3
TNFα	Tumor Necrosis Factor α
Treg	T Regulatory
TT	Total Therapy
VCAM-1	Vascular Cell Adhesion Molecule 1
VEGFA	Vascular Endothelial Growth Factor *A*
VEGFR2	Vascular Endothelial Growth Factor Receptor 2
VLA-4	Very Late Antigen-4
WNT-5a	Wnt Family Member 5a
